# Measurement of Daily Actions Associated With Mental Health Using the Things You Do Questionnaire–15-Item: Questionnaire Development and Validation Study

**DOI:** 10.2196/57804

**Published:** 2024-07-22

**Authors:** Madelyne A Bisby, Michael P Jones, Lauren Staples, Blake Dear, Nickolai Titov

**Affiliations:** 1 MindSpot Clinic MQ Health Macquarie University Macquarie Park Australia; 2 School of Psychological Sciences Macquarie University Macquarie Park Australia

**Keywords:** daily actions, depression, anxiety, psychometric, mental health, questionnaire, activities, goals, plans, healthy habits, habits, psychometric, treatment-seeking, treatment, confirmatory factor analysis, survey, adult, adult, assessment, digital psychology service, digital, psychology, depression symptoms, anxiety symptoms

## Abstract

**Background:**

A large number of modifiable and measurable daily actions are thought to impact mental health. The “Things You Do” refers to 5 types of daily actions that have been associated with mental health: healthy thinking, meaningful activities, goals and plans, healthy habits, and social connections. Previous studies have reported the psychometric properties of the Things You Do Questionnaire (TYDQ)–21-item (TYDQ21). The 21-item version, however, has an uneven distribution of items across the 5 aforementioned factors and may be lengthy to administer on a regular basis.

**Objective:**

This study aimed to develop and evaluate a brief version of the TYDQ. To accomplish this, we identified the top 10 and 15 items on the TYDQ21 and then evaluated the performance of the 10-item and 15-item versions of the TYDQ in community and treatment-seeking samples.

**Methods:**

Using confirmatory factor analysis, the top 2 or 3 items were used to develop the 10-item and 15-item versions, respectively. Model fit, reliability, and validity were examined for both versions in 2 samples: a survey of community adults (n=6070) and adults who completed an assessment at a digital psychology service (n=14,878). Treatment responsivity was examined in a subgroup of participants (n=448).

**Results:**

Parallel analysis supported the 5-factor structure of the TYDQ. The brief (10-item and 15-item) versions were associated with better model fit than the 21-item version, as revealed by its comparative fit index, root-mean-square error of approximation, and Tucker-Lewis index. Configural, metric, and scalar invariance were supported. The 15-item version explained more variance in the 21-item scores than the 10-item version. Internal consistency was appropriate (eg, the 15-item version had a Cronbach α of >0.90 in both samples) and there were no marked differences between how the brief versions correlated with validated measures of depression or anxiety symptoms. The measure was responsive to treatment.

**Conclusions:**

The 15-item version is appropriate for use as a brief measure of daily actions associated with mental health while balancing brevity and clinical utility. Further research is encouraged to replicate our psychometric evaluation in other settings (eg, face-to-face services).

**Trial Registration:**

Australian New Zealand Clinical Trials Registry ACTRN12613000407796; https://tinyurl.com/2s67a6ps

## Introduction

Depressive and anxiety disorders are highly prevalent [1-3]. Although effective psychological treatments exist [4], many people are unable or choose not to access care [5-7]. Important efforts have been made to reduce barriers to access, including remote treatment delivered via the internet [8] or through large-scale, low-intensity service models [9]. In addition, there is value in understanding the things people can do each day (ie, daily actions) to develop and maintain good mental health [10-12].

A broad range of actions, including physical activity [13], practicing gratitude and self-kindness [14], and social connectedness [15], have been argued to improve mental health. However, few studies have compared the benefits of different actions or explored the minimum frequency required to obtain psychological benefits. In a recent study, we explored the relationship between the frequency of performing different actions and mental health status (ie, depression, anxiety, and life satisfaction) in a large survey sample of community volunteers [12]. The actions included those identified by previous research as having a strong association with mental health (eg, physical activity and gratitude) and many others. Using survey development methodology [16], 5 different categories of daily actions (ie, factors) were found to have the strongest associations with mental health: healthy thinking (eg, treating oneself with respect), meaningful activities (eg, doing something enjoyable), goals and plans (eg, making plans and following through on them), healthy habits (eg, a consistent sleep-wake routine), and social connections (eg, having meaningful conversations).

As a result of the aforementioned study, a questionnaire including the 21 daily actions was developed, which comprised the 5 factors. The Things You Do Questionnaire (TYDQ)–21-item (TYDQ21) was found to be psychometrically valid and reliable, and higher scores on the TYDQ21 were associated with lower depression and anxiety symptoms in subsequent studies using treatment-seeking samples [17,18]. Furthermore, during an 8-week internet-based treatment for adults with depression or anxiety, increases in the frequency of TYDQ21 actions were highly correlated with decreases in depression and anxiety [17,18]. Across all studies, adults who reported doing the actions captured by the TYDQ21 at least on half of the days in each week reported significantly lower depression and anxiety symptoms than those who carried out the actions on less than half of the days in each week. This pattern was observed across all 5 types of actions [12,17,18].

Although relatively brief, the moderate length of the TYDQ21 limits its utility for regular administration during research or treatment trials. The primary purpose of this study is to determine whether the number of items in the TYDQ21 could be reduced while retaining strong psychometric properties. The secondary purpose of this study was to examine the factor structure in a larger sample of Australian adults who engaged with a national digital psychology service that provides free mental health care, funded by the Australian Government [7]. The performance of 10-item and 15-item versions were compared using the community sample of adults who completed an internet-based survey (n=6070) and a sample of adults in routine clinical care who completed an initial assessment at that service (n=14,878).

## Methods

### Study Design and Participants

A community sample (sample 1) was obtained from the original validation study [12]. Australian adults aged 18 years and older completed internet-based surveys on daily actions and mental health. Participants were recruited via social media and web newsletters from Australian mental health services; they were invited to participate in a study about daily activities and mental health. For this study, only those individuals with complete data for the TYDQ21 were included, and the assessments carried out in the original study [12] have been collapsed (n=6070).

The routine care sample (sample 2) includes adults who completed an initial assessment at the MindSpot Clinic between September 3, 2021, and May 26, 2022. The MindSpot Clinic is funded by the Australian Department of Health to provide remotely delivered psychological assessments and treatments to adults across Australia; clinical procedures and outcomes have been previously reported [7]. Eligibility criteria included being aged 18 years or older and being an Australian resident eligible for public health services. Only individuals who completed an initial assessment were included (n=14,878).

A subsample of the routine care sample (n=448) was considered for treatment responsivity analyses. This subgroup included those individuals who (1) completed the TYDQ during the initial assessment (between November 2021 and May 2022), and (2) completed at least 1 lesson of an internet-based treatment program for depression and anxiety. The Wellbeing Course is an evidence-based, 5-lesson, 8-week transdiagnostic treatment program designed to help adults manage depression and anxiety symptoms. Participants were offered weekly contact with a therapist to support them through the treatment. Further details regarding the Wellbeing Course can be found in the study by Titov et al [7].

### Ethical Considerations

The community sample was obtained from the study by Titov et al [7]. The study was approved by the Macquarie University Human Research Ethics Committee (5201700988). Deidentified data obtained from the MindSpot Clinic were analyzed for the treatment-seeking sample. This study is registered on the Australian New Zealand Clinical Trials Registry (ACTRN12613000407796). All participants provided informed consent. All data are stored securely, and participants did not receive payment or compensation for questionnaire completion.

### Measures

#### Things You Do Questionnaire

The TYDQ is a 21-item self-report measure of daily actions that are associated with mental health [12]. There are 5 factors: healthy thinking, meaningful activities, goals and plans, healthy habits, and social connections. Items are scored from 0 (not at all in the last week) to 4 (every day in the last week).

#### Patient Health Questionnaire-9

The Patient Health Questionnaire-9 (PHQ-9) is a self-report measure of depressive symptoms, consistent with a diagnosis of major depressive disorder [19]. Items are scored from 0 (not at all) to 3 (almost every day).

#### Generalized Anxiety Disorder 7-Item Questionnaire

The Generalized Anxiety Disorder 7-item questionnaire (GAD-7) is a self-report measure of anxiety symptoms, which captures symptoms consistent with generalized anxiety disorder [20]. Items are scored from 0 (not at all) to 3 (almost every day).

### Statistical Analysis

This study aimed to statistically confirm the previously published structure of the TYDQ21 and explore the psychometric properties of the 10-item and 15-item versions. Complete data were used for analyses at baseline, including factor analyses. When longitudinal data were used, missing data at midtreatment and posttreatment were handled using multiple imputation to replace missing values. Baseline scores (depression, anxiety, and daily actions) and lesson completion were considered predictors of missingness in the imputation model [21].

Confirmation for the number of latent variables was obtained via parallel analysis with the number determined from the number of factors where the observed eigenvalue was greater than the corresponding simulated value [22]. Confirmatory factor analysis was used to assess the fit of the originally published model in the new data and to evaluate the fit of revised versions. The model fit profile included a chi-square fit test *P* value of >.05, ratios of chi-square–df values of <5, a root-mean-square error of approximation of <0.05, comparative fit index of >0.90, and a Tucker-Lewis fit index of >0.90, based on published guidelines [23]. Configural, metric, and scalar invariance were examined across the community and routine care samples with the following benchmarks: root-mean-square error of approximation of <0.05, comparative fit index of >0.95, and Tucker-Lewis fit index of >0.95. CFA was implemented using MPlus (version 8) [24] and standardized loadings are reported throughout.

The TYDQ–10-item (TYDQ10) and –15-item (TYDQ15) versions were created by selecting those items with the highest factor loadings in the full version CFA model. The top 2 items for each factor were used to develop the TYDQ10, while the top 3 items were used to develop the TYDQ15. The items selected can be found in Table 1.

**Table 1:** Items included in the Things You Do Questionnaire–10- and –15-item versions and factor loadings for the 21-item version.

Item	Label	Factor	21-item version	15-item version	10-item version
1	I kept a healthy daily routine	Routine	0.75	✓	✓
2	I went to bed and woke up at a regular time	Routine	0.56	✓	
3	I treated myself with respect	Thoughts	0.71	✓	✓
4	Instead of worrying about the past, I focused on my preferred future	Thoughts	0.60		
5	I dealt with feelings of frustration or impatience in a healthy way	Thoughts	0.68	✓	
6	I socialized with positive people	Social	0.60	✓	
7	I talked about my day with a friend or family member	Social	0.77	✓	✓
8	I had a meaningful conversation with someone	Social	0.85	✓	✓
9	I spent time doing something I believed in	Goals	0.68		
10	I had a good laugh or did something fun	Activity	0.73		
11	I made a plan and stuck to it	Goals	0.67		
12	I had something to look forward to	Activity	0.74	✓	
13	I allowed myself to be less than perfect	Thoughts	0.44		
14	I prepared and ate a healthy meal	Routine	0.71	✓	✓
15	I did something that was very satisfying to me	Activity	0.76	✓	✓
16	I stopped myself from thinking unhelpful or unrealistic thoughts	Thoughts	0.65		
17	I kept a realistic perspective on things	Thoughts	0.73	✓	✓
18	I did something to help me achieve my goals	Goals	0.79	✓	
19	I did something to improve or maintain the quality of my life	Goals	0.84	✓	✓
20	I did something to help me live my ‘ideal’ life	Goals	0.82	✓	✓
21	I did something enjoyable	Activity	0.81	✓	✓

The variance in the TYDQ21 that is explained by the reduced (10- and 15-item) versions was evaluated by the model’s *R*^2^ value with the full version as the dependent variable and the reduced versions as the independent variables. The statistical significance of individual domain scores was assessed via nonparametric bootstrapping using 2000 bootstrap samples. Generalized estimating equations using a gamma log-link function and unstructured correlation matrix were used to examine responsivity to treatment change. We report the estimated marginal means and percentage change (95% CIs).

## Results

### Sample Characteristics

In the community sample, the mean age was 45.30 (SD 15.66) years, 63.06% (n=3766) of them were female, 53.05% (n=3168) of them lived in a capital city, and 58.94% (n=3520) of them were employed (see Multimedia Appendix 1). Half of the sample was married or in a de facto relationship (n=2979, 49.88%), and there was a wide range of education levels (eg, trade certificate [n=1738, 29.10%] and undergraduate degree [n=1725, 28.88%]). The sample was mostly Australian born (n=4860, 81.38%) and 30.27% (n=1808) of them had engaged with a mental health professional at the time of the survey or in the past (n=2831, 47.40%). Less than half of the sample reported clinical-level symptoms of depression (n=2655, 44.46%) or anxiety (n=2624, 43.94%) as indicated by a score of ≥10 on the PHQ-9 and GAD-7, respectively.

Participants in the treatment-seeking sample were slightly younger (mean 34.39, SD 13.16 years), primarily female (n=11,225, 75.45%), lived in a capital city (n=9083, 61.05%), and were married or in a de facto relationship (n=5564, 37.40%). Most participants were employed (n=9683, 65.08%), 41.73% (n=6208) of them had received a tertiary education, most of them (n=11,705, 78.67%) were born in Australia, and 41.75% (n=6212) of them had previously used mental health services. Unlike the community sample, the majority of participants in the treatment-seeking sample reported symptoms consistent with clinical levels of depression (n=11,210, 75.35%) or anxiety (n=9643, 64.81%).

### Parallel Analysis

In both samples, parallel analyses indicated that a 5-factor solution was the most appropriate.

### Confirmatory Factor Analysis

Confirmatory models were used to compare the fit of the TYDQ10 and TYDQ15 against the full TYDQ21 using both the community and treatment-seeking samples. A detailed summary of fit statistics can be found in Table 2. TYDQ15 was associated with superior model fit statistics than the TYDQ21 in both community and treatment-seeking samples. Similarly, the TYDQ10 was associated with a better model fit than the full TYDQ21. Minimal differences in model fit were seen for the TYDQ10 and TYDQ15. Configural, metric, and scalar invariance was appropriate for all TYDQ versions (see Multimedia Appendix 2).

**Table 2:** Confirmatory factor analyses of the 21-item, 15-item, and 10-item versions of the Things You Do Questionnaire in the community and treatment-seeking samples.

	Community				Treatment-seeking		
	21-item version	15-item version	10-item version	21-item version		15-item version	10-item version
Likelihood ratio^a^	<.001	<.001	<.001	<.001		<.001	<.001
Root-mean-square error of approximation	0.05	0.04	0.05	0.07		0.05	0.05
Confirmatory fit index	0.96	0.98	0.98	0.92		0.97	0.99
Tucker-Lewis fit index	0.95	0.97	0.97	0.91		0.96	0.97

^a^Refers to a significance test.

### Prediction of Original Factor Scores Using Reduced Items

Regression models examined the proportion of variance in the original factor scores, which could be explained by the reduced-item factors, as reported in Multimedia Appendix 3. Across both community and treatment-seeking samples, the TYDQ15 explained a higher amount of variance than the TYDQ10. For example, the variance explained by the TYDQ10 and TYDQ15 of the “Healthy Thinking” factor using the community sample (*R*^2^=0.77 vs 0.86) and treatment-seeking sample (*R*^2^=0.77 vs 0.85).

### Internal Consistency

In the community sample, internal consistency was excellent for the TYDQ21 (α=0.93) and TYDQ15 (α=0.91) and good for the TYDQ10 (α=0.87). A similar pattern was found in the treatment-seeking sample. Internal consistency was excellent for the TYDQ21 (α=0.92) and TYDQ15 (α=0.90) and slightly lower for the TYDQ10 (α=0.86).

### Construct Validity

Bivariate correlations between the total and factor scores on the TYDQ versions and psychological measures are reported in Table 3. Across both community and treatment-seeking samples, the full TYDQ21 showed moderate correlations with the PHQ-9 (*r*=–0.30 to –0.63) and weak to moderate correlations with the GAD-7 (*r*=–0.13 to –0.56). Both abbreviated versions of the TYDQ demonstrated weaker correlations with psychological measures, although there were no marked differences between the 2 versions. The PHQ-9 was moderately correlated with the total and factor scores of the TYDQ10 and TYDQ15 (*r*=–0.27 to –0.56) in the community sample. These correlations were slightly, but not meaningfully, higher in the treatment-seeking sample (*r*=–0.36 to –0.63). The GAD-7 demonstrated weak to moderate correlations with the TYDQ10 and TYDQ15 total and factor scores in the community sample (*r*=–0.10 to –0.42) and treatment-seeking sample (*r*=–0.27 to –0.58).

**Table 3:** Correlations between the Things You Do Questionnaire (TYDQ) versions and psychological measures in the community and treatment-seeking samples.

		Community			Treatment-Seeking	
		PHQ-9^a^	GAD-7^b^	PHQ-9		GAD-7
**21-item version**						
	Total	–0.63	–0.54	–0.56		–0.36
	Healthy thinking	–0.59	–0.58	–0.49		–0.42
	Meaningful activity	–0.56	–0.48	–0.43		–0.26
	Goals and plans	–0.49	–0.38	–0.45		–0.26
	Healthy habits	–0.53	–0.40	–0.50		–0.28
	Social connection	–0.40	–0.31	–0.30		–0.13
**15-item version**						
	Total	–0.56	–0.34	–0.63		–0.53
	Healthy thinking	–0.52	–0.42	–0.60		–0.58
	Meaningful activity	–0.44	–0.27	–0.56		–0.47
	Goals and plans	–0.41	–0.24	–0.44		–0.35
	Healthy habits	–0.50	–0.28	–0.53		–0.40
	Social connection	–0.30	–0.12	–0.40		–0.31
**10-item version**						
	Total	–0.54	–0.33	–0.62		–0.52
	Healthy thinking	–0.51	–0.41	–0.59		–0.57
	Meaningful activity	–0.40	–0.25	–0.52		–0.45
	Goals and plans	–0.37	–0.23	–0.44		–0.36
	Healthy habits	–0.45	–0.25	–0.49		–0.37
	Social connection	–0.27	–0.10	–0.36		–0.27

^a^PHQ-9: Patient Health Questionnaire-9.

^b^GAD-7: Generalized Anxiety Disorder 7-item questionnaire.

### Responsivity to Treatment

Scores on all TYDQ versions increased from initial assessment to posttreatment (see Table 4). The largest percentage change was observed on the full TYDQ21 (39%, 95% CI 32%-46%) with slightly lower percentage improvements observed on the abbreviated TYDQ15 (35%, 95% CI 28%-42%) and TYDQ10 (32%, 95% CI 25%-39%).

**Table 4:** Estimated marginal means and percentage change over treatment for Things You Do Questionnaire versions.

	21-item version	15-item version	10-item version
Application, mean (SE)	27.82 (0.62)	20.71 (0.46)	14.17 (0.33)
Pretreatment, mean (SE)	32.45 (0.67)	23.95 (0.50)	16.23 (0.35)
Midtreatment, mean (SE)	36.78 (0.63)	26.69 (0.47)	17.96 (0.33)
Posttreatment, mean (SE)	38.80 (0.77)	27.96 (0.56)	18.69 (0.38)
Percentage change (95% CI)	39 (32-46)	35 (28-42)	32 (25-39)

## Discussion

This study developed and evaluated a brief version of the TYDQ21 [12], a measure of daily actions associated with mental health. The questionnaire assesses how often individuals perform 5 different types of healthy actions, referred to as the “Things You Do.” These 5 domains include healthy thinking, meaningful activities, goals and plans, healthy habits, and social connections. The abbreviated questionnaire versions included the top 2 items (TYDQ10) or 3 items (TYDQ15) with the highest loading on each of the 5 factors. This approach enabled us to retain and replicate the original 5-factor structure while assessing the psychometric properties of these 2 brief questionnaires. Overall, the TYDQ15 emerged as the preferred abbreviation of the full TYDQ21. Compared to the TYDQ10, the TYDQ15 showed acceptable model fit, explained a larger proportion of variance on the full 21-item version, and had slightly higher internal consistency. Both the TYDQ15 and TYDQ10 were responsive to treatment and increased over time; however, improvements on the TYDQ15 more closely resembled that of the full 21-item measure. Therefore, although both brief adaptations appear psychometrically sound, the current study recommends the use of the TYDQ15 to maintain a balance between brevity and clinical utility.

Unlike on the TYDQ21, each of the 5 factors on the TYDQ15 include an equal number of items (ie, 3 items per factor). The number of items per factor ranged from 3 to 7 on the TYDQ21 [12]; as a result, the total score was more heavily weighted toward the factors with the most items (ie, healthy thinking, goals, and plans). By removing the additional items from overrepresented factors, the TYDQ15 total score provides a more appropriate overall average estimate of how often individuals are performing the “Things You Do” actions. It is possible that even the distribution of the 5 factors within the model resulted in the superior model fit observed in this study. As a psychometric evaluation study, we examined longitudinal changes in total but not factor scores on the TYDQ versions. Recent evidence suggests that the 5 factors on longer versions of the TYDQ change differently across treatment; indeed, slightly larger pre-post increases have been observed for the healthy thinking (*d*=0.58), meaningful activities (*d*=0.62), and goals and plans (*d*=0.62) factors than for the healthy routines (*d*=0.44) and social connections (*d*=0.29) [25]. Further work is needed to explore longitudinal changes in the frequency of daily actions over treatment and to delineate the relative contributions of treatment versus nonspecific factors to these changes.

This study is not without its limitations. We attempted to make our findings as generalizable as possible by conducting our analyses in a large community sample (n>6000) and large treatment-seeking sample (n>14,000). However, these samples are limited by their inclusion of only Australian adult participants. In addition, the demographics of the treatment-seeking sample are somewhat skewed (eg, mostly female). Further research is needed to replicate our psychometric evaluation of the TYDQ15 in other demographic groups—this is critical for understanding the influence of contextual factors on the association between daily actions and mental health. Second, the TYDQ was optional to complete during the initial assessment for the treatment-seeking sample, resulting in item- and scale-level missingness. This decision was made to reduce questionnaire burden considering that participants were signing up for a health service rather than directly participating in a research trial. Third, this study did not have access to a sample in which the TYDQ is administered regularly in the absence of treatment, and this is an avenue for future research.

This study provides a psychometric evaluation of the TYDQ15 as a measure of daily actions associated with mental health. The abbreviated 15-item version was associated with acceptable reliability, validity, model fit, and responsivity to treatment. The TYDQ15 is more amenable to regular administration during psychological interventions (eg, on a weekly or fortnightly basis) than the TYDQ21. By retaining 3 items per factor, the clinical utility of the questionnaire is largely retained. Indeed, there is still scope for practitioners to review how often individuals are performing different types of daily actions and then supporting individuals to increase how often they perform specific actions based on this personalized feedback. For instance, regular administration of the TYDQ15 may assist practitioners to support their patients in identifying barriers to fostering social relationships or reducing social isolation based on their social connections score or to focus on reducing self-critical thinking or developing emotion regulation skills based on their healthy thinking score. This monitoring and feedback approach could be taken alongside not only standard psychological treatments, but also low-intensity interventions carefully designed to support individuals to increase how often they are doing the specific daily actions associated with mental health. Future research may explore the potential of action-based interventions, together with routine monitoring of daily action frequency using the TYDQ15, to improve mental health using fewer resources than traditional psychological treatments. Our findings suggest that the TYDQ15 is an appropriate measure of daily actions that are associated with mental health and may hold utility as a clinical resource.

## Appendix

Multimedia Appendix 1 Clinical and demographic characteristics of community and treatment-seeking samples.

Multimedia Appendix 2 Measurement invariance in community and treatment-seeking samples.

Multimedia Appendix 3 Variance explained (R2) in the 21-item measure by the briefer versions in the community and treatment-seeking samples.

## Abbreviations

CFA: confirmatory factor analysis
GAD-7: Generalized Anxiety Disorder 7-item questionnaire
PHQ-9: Patient Health Questionnaire-9
TYDQ: Things You Do Questionnaire
TYDQ10: Things You Do Questionnaire–10-item
TYDQ15: Things You Do Questionnaire–15-item
TYDQ21: Things You Do Questionnaire–21-item

## Data Availability

The data set from the original survey is available from the senior author (NT) upon reasonable request. Due to the confidential nature of the treatment-seeking sample, the data are not available for sharing.

## References

[1] Moreno-Agostino D, Wu Y, Daskalopoulou C, Hasan MT, Huisman M, Prina M (2021). Global trends in the prevalence and incidence of depression: a systematic review and meta-analysis. J Affect Disord.

[2] Richter D, Wall A, Bruen A, Whittington R (2019). Is the global prevalence rate of adult mental illness increasing? Systematic review and meta-analysis. Acta Psychiatr Scand.

[3] Yang X, Fang Y, Chen H, Zhang T, Yin X, Man J, Yang L, Lu M (2021). Global, regional and national burden of anxiety disorders from 1990 to 2019: results from the Global Burden of Disease Study 2019. Epidemiol Psychiatr Sci.

[4] Cuijpers P, Quero S, Noma H, Ciharova M, Miguel C, Karyotaki E, Cipriani A, Cristea IA, Furukawa TA (2021). Psychotherapies for depression: a network meta-analysis covering efficacy, acceptability and long-term outcomes of all main treatment types. World Psychiatry.

[5] Andrade LH, Alonso J, Mneimneh Z, Wells JE, Al-Hamzawi A, Borges G, Bromet E, Bruffaerts R, de Girolamo G, de Graaf R, Florescu S, Gureje O, Hinkov HR, Hu C, Huang Y, Hwang I, Jin R, Karam EG, Kovess-Masfety V, Levinson D, Matschinger H, O'Neill S, Posada-Villa J, Sagar R, Sampson NA, Sasu C, Stein DJ, Takeshima T, Viana MC, Xavier M, Kessler RC (2013). Barriers to mental health treatment: results from the WHO World Mental Health surveys. Psychol Med.

[6] Coombs NC, Meriwether WE, Caringi J, Newcomer SR (2021). Barriers to healthcare access among U.S. adults with mental health challenges: a population-based study. SSM Popul Health.

[7] Titov N, Dear BF, Nielssen O, Wootton B, Kayrouz R, Karin E, Genest B, Bennett-Levy J, Purtell C, Bezuidenhout G, Tan R, Minissale C, Thadhani P, Webb N, Willcock S, Andersson G, Hadjistavropoulos HD, Mohr DC, Kavanagh DJ, Cross S, Staples LG (2020). User characteristics and outcomes from a national digital mental health service: an observational study of registrants of the Australian MindSpot Clinic. Lancet Digital Health.

[8] Andersson G, Titov N, Dear BF, Rozental A, Carlbring P (2019). Internet-delivered psychological treatments: from innovation to implementation. World Psychiatry.

[9] Wakefield S, Kellett S, Simmonds-Buckley Melanie, Stockton D, Bradbury A, Delgadillo J (2021). Improving Access to Psychological Therapies (IAPT) in the United Kingdom: a systematic review and meta-analysis of 10-years of practice-based evidence. Br J Clin Psychol.

[10] Donovan RJ, Anwar-McHenry J (2016). Act-belong-commit: lifestyle medicine for keeping mentally healthy. Am J Lifestyle Med.

[11] Santini ZI, Nelausen MK, Kusier AO, Hinrichsen C, Schou-Juul F, Madsen KR, Meilstrup C, Donovan RJ, Koushede V, Nielsen L (2022). Impact evaluation of the “ABCs of Mental Health” in Denmark and the role of mental health-promoting beliefs and actions. MHSI.

[12] Titov N, Dear BF, Bisby MA, Nielssen O, Staples LG, Kayrouz R, Cross S, Karin E (2022). Measures of daily activities associated with mental health (Things You Do Questionnaire): development of a preliminary psychometric study and replication study. JMIR Form Res.

[13] Teychenne M, White RL, Richards J, Schuch FB, Rosenbaum S, Bennie JA (2020). Do we need physical activity guidelines for mental health: what does the evidence tell us?. MENPA.

[14] Bohlmeijer ET, Kraiss JT, Watkins P, Schotanus-Dijkstra M (2020). Promoting gratitude as a resource for sustainable mental health: results of a 3-armed randomized controlled trial up to 6 months follow-up. J Happiness Stud.

[15] Wickramaratne P, Yangchen T, Lepow L, Patra B, Glicksburg B, Talati A, Adekkanattu P, Ryu E, Biernacka J, Charney A, Mann J, Pathak J, Olfson M, Weissman M (2022). Social connectedness as a determinant of mental health: a scoping review. PLoS One.

[16] Boateng GO, Neilands TB, Frongillo EA, Melgar-Quiñonez Hugo R, Young SL (2018). Best practices for developing and validating scales for health, social, and behavioral research: a primer. Front Public Health.

[17] Bisby MA, Titov N, Dear BF, Karin E, Wilhelms A, Nugent M, Hadjistavropoulos HD (2022). Examining change in the frequency of adaptive actions as a mediator of treatment outcomes in internet-delivered therapy for depression and anxiety. J Clin Med.

[18] Bisby MA, Dear BF, Karin E, Fogliati R, Dudeney J, Ryan K, Fararoui A, Nielssen O, Staples LG, Kayrouz R, Cross S, Titov N (2023). An open trial of the Things You Do Questionnaire: changes in daily actions during internet-delivered treatment for depressive and anxiety symptoms. J Affect Disord.

[19] Kroenke K, Spitzer RL, Williams JBW (2001). The PHQ-9: validity of a brief depression severity measure. J Gen Intern Med.

[20] Spitzer RL, Kroenke K, Williams JBW, Löwe Bernd (2006). A brief measure for assessing generalized anxiety disorder: the GAD-7. Arch Intern Med.

[21] Karin E, Crane MF, Dear BF, Nielssen O, Heller GZ, Kayrouz R, Titov N (2021). Predictors, outcomes, and statistical solutions of missing cases in web-based psychotherapy: methodological replication and elaboration study. JMIR Ment Health.

[22] Hayton JC, Allen DG, Scarpello V (2016). Factor retention decisions in exploratory factor analysis: a tutorial on parallel analysis. Organ Res Methods.

[23] Schermelleh-Engel K, Moosbrugger H, M✓ller H (2003). Evaluating the fit of structural equation models: tests of significance and descriptive goodness-of-fit measures. Methods Psychol Res Online.

[24] Muthén L, Muthén B (2017). Mplus: statistical analysis with latent variables. User's guide.

[25] Bisby M, Staples L, Dear B, Titov N (2024). Examining change in the frequency of adaptive actions as a mediator of treatment outcomes in internet-delivered therapy for depression and anxiety. J Form Res under review.

