# Supplementary material for: Measurement of Daily Actions Associated With Mental Health Using the Things You Do Questionnaire–15-Item: Questionnaire Development and Validation Study
Source: JMIR Form Res. 2024 Jul 22;8:e57804. doi: 10.2196/57804 (PMC11301108; doi:10.2196/57804)
Supplement: Multimedia Appendix 1 [file formative_v8i1e57804_app1.docx]

**Multimedia Appendix 1.** Clinical and demographic characteristics of community and treatment-seeking samples.

|  | **Community**  **n (%)** | | **Treatment-Seeking**  **n (%)** |
| --- | --- | --- | --- |
| **Age** (M, SD) | 45.30 (15.66) | | 34.39 (13.16) |
| **Gender** |  | |  |
| Male | 2181 (37) | | 3473 (23) |
| Female | 3766 (63) | | 11225 (75) |
| Other | 25 (0) | | 180 (1) |
| **Location** |  | |  |
| Capital city or surrounds | 3168 (53) | | 9083 (61) |
| Other urban region | 1460 (24) | | 3073 (21) |
| Rural or remote region | 1344 (23) | | 2722 (18) |
| **Marital Status** |  | |  |
| Married or de facto | 2979 (50) | | 5564 (37) |
| Other | 2993 (50) | | 9320 (63) |
| **Employment** |  | |  |
| Employed | 3520 (59) | | 9683 (65) |
| Student | 508 (9) | | 1823 (12) |
| Unemployed | 477 (8) | | 1415 (10) |
| Home duties | 265 (4) | | 947 (6) |
| Retired | 876 (15) | | 507 (3) |
| Disability support/ benefit | 325 (5) | | 503 (3) |
| **Education** |  | |  |
| High school or less | 1005 (17) | | 4649 (31) |
| Trade certificate/diploma | 1738 (29) | | 4021 (27) |
| Undergraduate degree | 1725 (29) | | 3527 (24) |
| Postgraduate degree | 1504 (25) | | 2681 (18) |
| **Born in Australia** |  | |  |
| No | 1112 (19) | | 3173 (21) |
| Yes | 4860 (81) | | 11705 (79) |
| **Baseline Symptoms** |  | |  |
| PHQ-9 ≥ 10 | 2655 (44) | | 11210 (75) |
| GAD-7 ≥ 10 | 2624 (43) | | 9643 (65) |
| **Previous Use of Mental Health Professionals** | |  | |
| Never | 1333 (22) | | 5730 (39) |
| Previous | 2831 (47) | | 6212 (42) |
| Current | 1808 (30) | | 2936 (20) |
| *Note.* Demographic information available for 5,972 participants (98.4% of total sample). | | | |
