# Supplementary material for: Measurement of Daily Actions Associated With Mental Health Using the Things You Do Questionnaire–15-Item: Questionnaire Development and Validation Study
Source: JMIR Form Res. 2024 Jul 22;8:e57804. doi: 10.2196/57804 (PMC11301108; doi:10.2196/57804)
Supplement: Multimedia Appendix 2 [file formative_v8i1e57804_app2.docx]

**Multimedia Appendix 2**. Confirmatory factor analyses of the 21, 15 and 10 item versions comparing community and treatment-seeking samples.

|  | **21-item** | | | **15-item** | | | **10-item** | | |
| --- | --- | --- | --- | --- | --- | --- | --- | --- | --- |
|  | **RMSEA** | **CFI** | **TLI** | **RMSEA** | **CFI** | **TLI** | **RMSEA** | **CFI** | **TLI** |
| **Configural** | 0.05 | 0.99 | 0.98 | 0.05 | 0.97 | 0.96 | 0.06 | 0.93 | 0.92 |
| **Metric** | 0.05 | 0.99 | 0.98 | 0.05 | 0.97 | 0.96 | 0.06 | 0.93 | 0.92 |
| **Scalar** | 0.06 | 0.97 | 0.96 | 0.05 | 0.96 | 0.95 | 0.06 | 0.92 | 0.92 |
