# Supplementary material for: Measurement of Daily Actions Associated With Mental Health Using the Things You Do Questionnaire–15-Item: Questionnaire Development and Validation Study
Source: JMIR Form Res. 2024 Jul 22;8:e57804. doi: 10.2196/57804 (PMC11301108; doi:10.2196/57804)
Supplement: Multimedia Appendix 3 [file formative_v8i1e57804_app3.docx]

**Multimedia Appendix 3**. Variance explained (R^2^) in the 21-item measure by the briefer versions in the community and treatment-seeking samples.

|  | **Community** | | **Treatment-seeking** | |
| --- | --- | --- | --- | --- |
|  | **15-item** | **10-item** | **15-item** | **10-item** |
| **Healthy Thinking** | 0.86 | 0.79 | 0.85 | 0.77 |
| **Meaningful Activities** | 0.96 | 0.87 | 0.95 | 0.86 |
| **Goals & Plans** | 0.90 | 0.83 | 0.89 | 0.82 |
| **Healthy Habits** | -- | 0.85 | -- | 0.85 |
| **Social Connection** | -- | 0.90 | -- | 0.89 |
| *Note.* R^2^ reported. |  |  |  |  |
